# Affirming and Inclusive Care Training for Medical Students and Residents to Reduce Health Disparities Experienced by Sexual and Gender Minorities: A Systematic Review

**DOI:** 10.1089/trgh.2021.0148

**Published:** 2023-07-28

**Authors:** Robert Lyle Cooper, Aramandla Ramesh, Asa E. Radix, Jayne S. Reuben, Paul D. Juarez, Cheryl L. Holder, Allyson S. Belton, Katherine Y. Brown, Leandro A. Mena, Patricia Matthews-Juarez

**Affiliations:** ^1^ Department of Family and Community Medicine, Cancer Biology, Neuroscience & Pharmacology, Meharry Medical College, Nashville, Tennessee, USA.; ^2^ Department of Biochemistry, Cancer Biology, Neuroscience & Pharmacology, Meharry Medical College, Nashville, Tennessee, USA.; ^3^ National Center for Medical Education Development and Research, Meharry Medical College, Nashville, Tennessee, USA.; ^4^ Callen-Lorde Community Health Center in New York, New York, New York, USA.; ^5^ Department of Biomedical Sciences, Texas Agricultural and Mechanical University College of Dentistry, Dallas, Texas, USA.; ^6^ Department of Family Medicine at Herbert Wertheim College of Medicine, Florida International University, Miami, Florida, USA.; ^7^ Satcher Health Leadership Institute at Morehouse School of Medicine, Atlanta, Georgia, USA.; ^8^ Division of STD Prevention, National Center for HIV/AIDS, Viral Hepatitis, STD, and TB Prevention, Centers for Disease Control and Prevention, Atlanta, Georgia, USA.

**Keywords:** affirming care, gender and sexual minorities, graduate medical education, LGBTQ, medical education, undergraduate medical education

## Abstract

**Purpose:**

Providing inclusive and comprehensive gender-affirming care is critical to reducing health disparities (gaps in care) experienced by sexual and gender minorities (SGM). Currently, little is known about how medical students and residents are being trained to address the health needs of SGM persons or of the most effective methods.

**Methods:**

We conducted a systematic review of the research literature from 2000 to 2020 on the effectiveness of teaching medical students and residents on knowledge, attitudes, and skills in addressing the health of SGM persons and the strength of the research sample, design, and methods used.

**Results:**

We identified a total of 36 articles that assessed the impact of medical student and resident education on knowledge, comfort, attitudes, confidence, and skills in working with SGM patients. All studies utilized quasi-experimental designs, and found efficacious results. No study examined the impact of training on patient outcomes.

**Conclusion:**

Future studies will need to be powered and designed to assess the impact of training on patient outcomes.

## Introduction

Sexual and gender minorities (SGM) experience a higher burden of health disparities compared to their heterosexual/cisgender counterparts (we use the term SGM throughout to represent all the acronyms used to represent lesbian, gay, bisexual, transgender, and queer people).^1^ Some of the poorer health outcomes observed in SGM populations appear to be associated with increased prevalence of bullying, emotional distress, alcohol and other drug use, intimate partner violence, sexually transmitted infections, including human immunodeficiency virus, and cancer.^1–10^ In addition, transgender people, in particular, experience unique barriers to receiving both routine and gender-affirming health care such as acquiring hormones and gender-affirming surgeries.^11–15^ Differences in access and utilization of care by patients who identify as sexual or gender minorities and the stress arising both from implicit and explicit bias, may be tied to discrimination as well as a limited knowledge of their specific needs or a lack of experience during medical training or practice.^16–23^

A survey of 132 medical schools in the United States found the median time of training medical students in working with lesbian, gay, bisexual, and transgender (LGBT) patients is 5 h.^24^ The barriers to implementing affirming training for SGM patients are at least partially driven by a lack of expertise among medical education faculty and preceptors.^1^ This is true of undergraduate medical education (UME), and even more pronounced in graduate medical education (GME).^25–27^ However, in 2007, the Association of American Medical Colleges (AAMC) published a report and provided recommendations acknowledging the need for medical students to receive additional training to more effectively manage the care of SGM patients to ensure the provision of “excellent, comprehensive care.” Furthermore, the AAMC report^28^ and two reviews on the status of medical student training^22,23^ noted the importance of affirming care training to reduce the disparities faced by this population.

Inclusive care for gender-diverse patients and gender-affirming care for transgender patients are designed to improve the health outcomes of SGM persons. Both approaches affirm the identities of SGM by making the health care system a more welcoming and inclusive environment.

## Methods

We conducted a systematic review using PRISMA guidelines of educational interventions to identify original studies that focused on education to increase knowledge and comfort, as well as improve the attitudes, confidence, and skills of medical students and residents working with SGM patients.

### Search strategy

We conducted database searches of Google Scholar, PubMed, OVID, ERIC, SCOPUS, Web of Science, CINAHL, PsycInfo, and MedED Portal, between 2000 and 2020 using Covidence software (Covidence Org, Melbourne, Australia). The full search strings for each database are provided in Table 1. All searches included some combination of the following terms (words used to represent affirming care separated by OR) AND (medical students OR residents OR medical education OR training OR curriculum). To ensure the most up-to-date articles were included in the search, we established a system within each database to alert the team to any new article published since the end of the active search.

**Table 1.** Systematic Review Search Terms

	Concept 1	Concept 2	Concept 3
OR	Lesbian	Medical Student	Training
OR	Gay	Internship	Lecture
OR	Homosexual	Residency	Small group discussion
OR	Homosexuality	Internship and Residency	OSCE
OR	Bisexual	House Staff	Course
OR	Bisexuality	Professional Education	Teaching
OR	Queer	Medical Education	Curriculum
OR	Questioning	Trainee	Sexual History
OR	Non-Heterosexual	Learner	Sexual Health
OR	Sexual Minorities		
OR	Transgender Persons		
OR	LGBT		
OR	Sexual Orientation		

### Eligibility criteria and study selection

A total of 27,090 articles were identified initially. This number was reduced to 21,063 using EndNote, to identify all duplicates and materials not published in peer-reviewed journals (i.e., book chapters, serials, etc.). The remaining article abstracts were reviewed using the following inclusion criteria: (a) published between January 2000 and June 2020; (b) designed as a research intervention of medical education/curriculum; (c) reported quantitative or qualitative results of the effect of the educational interventions; (d) published in English; and (e) targeted medical students and/or resident physicians. The exclusion criteria used for screening the abstracts were as follows: (a) articles that focused on continuing education in gender-affirming care for health care provider and (b) articles that described gender-affirming care curriculum without implementation.

Initial screening of all databases generated a total of 27,090 articles: Google Scholar (71), PubMed (745), OVID (9468), ERIC (108), SCOPUS (7651), Web of Science (5713), CINAHL (3001), PsycInfo (294), and MedEd Portal (39). After 4654 duplicates were removed, 22,436 articles remained in the search. One thousand three hundred seventy-three records were further excluded because the abstracts were from books (840), book chapters (431), and conference proceedings (102). The remaining 21,063 records were subjected to title and abstract review, which eliminated 21,015 articles, resulting in a total of 44 (48) articles.

**FIG. 1.**
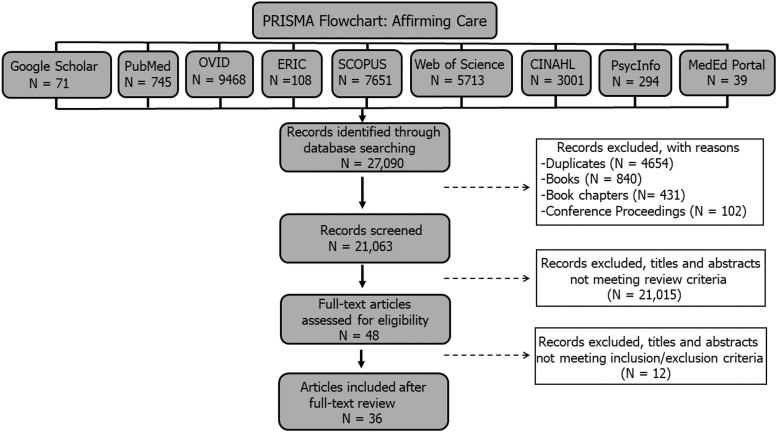
Schematic of PRISMA flowchart used for screening of affirming care and inclusive care literature.

At this stage of the review, 12 additional articles were removed because they either lacked a focus on medical education or did not include an evaluation of the described educational interventions in the study. After the full text review, 36 articles were left to be included in this systematic review. Figure 1 provides a PRISMA diagram of each step in our systematic review process.

### Data extraction

Data were extracted from all articles included: subject description, study design, sample/sample size, educational intervention, and intended outcome, as well as a summary of the findings and conclusions (all data^29–64^ are summarized in Table 2).

**Table 2.** Curricular Interventions for Affirming and Inclusive Care Training for Medical Students and Residents

Quality score	Source	Participants	Design	Intervention	Findings	Conclusions
2	Anderson et al.^29^	Included 349 osteopath medicine second year students (2 campuses 160+189) enrolled in an internal medicine program at the time of didactic sessions	Emailed pre-test and post-test surveys assessed the students' attitudes toward treating GSM patients and medical knowledge of GSM populations.	2 h of content was delivered over a 10-week curriculum regarding LGBT health. Content includes self-directed learning module addressing attitudes about and knowledge of GSM, as well as sexual history taking, and culminated in a standardized patient interview.	Clinical knowledge improved. However, student attitudes regarding GSM patients did not indicate significant change post-training.	A single didactic module may be insufficient in changing attitude scores. Further interventions need to include mixed didactics model comprising small group sessions, lectures, and clinical exposure to GSM patient(s).
2	Arora et al.^30^	Included 81 MS and 50 GPs, 57 internal medicine physicians in Australia.	Mixed-Method study. Pre- and post-lecture survey assessed student capacity to deliver transgender health care.	An 1-h didactic educational session regarding transgender health was provided.	Confidence in assisting adolescents requesting transition significantly improved post-training, as well as understanding of safety/risk profile of hormonal and surgical treatment. Belief that gender identity has a biological basis did not change from pre- to post-evaluation.	Knowledge increase was affected by a 1-h lecture, while beliefs were not. Didactic pedagogy may not be effective in changing trainee beliefs.
3	Bakhai et al.^31^	120 first year MS completed a pre-test evaluation, and 78 completed a post-test survey.	Pre- and post-test evaluation of sexual history taking with standardized GSM patients.	Training on taking sexual histories with SGM included a 14-min lecture, 35-min standardized patient interview, and a 20-min group debrief.	Participants reported improved knowledge, perceived preparedness, and satisfaction after receiving the training. Qualitative responses indicated greater awareness of bias and greater comfort taking sexual histories with GSM patients.	The brief standardized patient training was successful in improving sexual history taking. More training is necessary to improve overall work with GSM patients.
3	Bakhai et al.^32^	42 M3 and M4 students enrolled in a pediatric clerkship	Pre-test and post-test surveys that assessed the students' communication skills toward treating SGM youth.	An 8-week required pediatric clerkship was delivered. It included conducting a HEADSS exam with an SGM patient, as well as being in a clinical rotation being supervised by pediatricians experienced in treating SGM.	A significant change in student knowledge of the objective-based skills, and comfort and sense of preparedness in counseling adolescents questioning their sexual orientation before and after receiving the intervention were noted. Students noted the training also brought a greater awareness of the importance of specific health concerns that affect SGM youth.	The intervention helped the development of key communication skills required for students to provide competent and nonjudgmental care for SGM patients. Students expressed the need for more training from experienced physicians in handling sex orientation questions in clinical settings.
3	Berenson et al.^33^	123 second year MS.	Pre- and post-test single-group design, assessing self-confidence in working with transgender patients. The pre-test included a survey of campus climate regarding transphobia, and the post-test included satisfaction ratings for each element of the training.	Training included a didactic session on disparities experienced by transgendered patients, as well as training on office-based masculinizing/feminizing therapies; small group video viewing of poor and competent approaches to work with transgender patients; and a patient panel of transgender community members.	There were very few instances of reported transphobic behavior on campus. Scores rose significantly on all knowledge and confidence measures. Finally, satisfaction was reported as high for all elements of the training, with the patient panel being rated as the most helpful.	Patient panels are a particularly effective means for training MS to work with the transgender population. Involving GSM identified MS in the development of the training was seen as helpful. The training evaluated in this study built on previous one-year medical training, leading to review of and addition to additional material.
2	Braun et al.^34^	46 general medicine, dentistry, advanced practice nursing, and physical therapy students.	Pre-test, and immediate and 3 months post-test assessing transphobia (validated) and knowledge regarding affirming care.	10-h elective Transgender Health course was delivered. Topics included terminology, epidemiology, psychiatric, gynecological, urological and primary care, as well as policy and the history of transgender care. No discussion of pedagogy was given.	Transphobia decreased significantly at immediate post-test. Knowledge of cultural competence, health disparities, and state and federal policy did increase, but not significantly.	Knowledge, attitudes, and beliefs were amenable to change through the course. High pre-test scores may have limited the affect of the course. The sample for the 3-month post-test was too small to assess differences.
2	Calzo et al.^35^	Leadership Education in Adolescent Health fellows provided the training in 2013, 2015, and 2016 respectively. The interdisciplinary groups included medicine, nutrition, social work, and psychology students.	Formative evaluation involving pre- and post-test surveys as well verbal feedback were collected from each cohort across 3 years. Findings were used to modify the content over the 3-year evaluation period.	Four 1–3 h modules on GSM were provided. Topics included cultural humility; sexual orientation health disparities; the effect of family support/rejection; gender dysphoria and transgender youth; and advocacy and leadership. Small group exercises, case reviews, and self-directed learning were all used to convey the topics.	In 2013, data were collected on course satisfaction, and yielded high scores on several Likert scale items. In 2014, confidence of the participants was measured at pre- and post-test. Confidence slightly decreased in skills, while knowledge of available resources rose from pre- to post-test. The 2015 and 2016 data collection mirrored the 2013 data collection, and satisfaction ratings were again high.	Cultural humility was a concept identified as critical to successful training. The authors posit that the reduction in confidence in skills to work with this population may have resulted from recognizing what students did not know. Future study might include a larger sample and standardized measures of learning.
3	Cherabie et al.^36^	163 individuals (faculty, students, and residents) completed the initial survey, and 115 (70.6%) completed the post-intervention survey.	A pre-intervention and two post-intervention surveys (one after the session and the other 90 days post-intervention) were given to assess attitudes, comfort level, knowledge, and beliefs regarding the treatment of transgendered persons and associated health concerns.	1-h-long didactic lecture and presentations were provided. The didactic lecture included educational information on transgender health and presentations by transgender persons having completed transition from male to female, and female to male.	The intervention contributed to a positive and also significant increase in attitudes, comfort levels, and knowledge in regard to transgender health issues. There was no significant change in beliefs. The 90 days post-intervention did not show a significant increase in the above-mentioned criteria.	The positive change in attitudes, comfort, and knowledge about transgender health issues found at immediate post-test assessment is promising; however, these gains were not sustained at 90 days post-training, suggesting ongoing training may be needed to sustain initial gains.
3	Click et al.^37^	138 out of 140 MS completed the pre-test and immediate post-test, and 85 completed the 2-month follow-up data collection.	Pre-test and immediate post-test assessing student comfort with transgender patients, perception of knowledge, preference to treat transgender versus cisgender patients, knowledge of transgender care standards, and understanding of gender expression. Immediate post-test was followed by a 2 month post-intervention assessment	Content was delivered through a half-day “integrated grand round” session. These sessions included didactics followed by small group discussions, on planning a patient interview, creating safe environments, overcoming bias, and building a health care team. Finally a transgender patient participated in a question and answer session.	Student knowledge of transgender care standards improved significantly from pre-test to immediate post-test, as did student comfort working with transgender patients. However, understanding of gender expression did not improve.	Joint session planning between faculty and students was noted as an important factor in the module success. While knowledge improved significantly there is still room for improvement in this area. Importantly, at the 2 month follow-up student comfort increases remained as did the belief that providing hormone therapy for transgender patients was appropriate.
3	Cooper et al.^38^	63 of the 180 year-3 MS approached for this study completed the retrospective pre-/post-test survey.	A retrospective pre-/post-survey was conducted to assess how social determinants of health impact the care of GSM patients.	A 1-h didactic lecture in a traditional classroom setting was given by a content expert in GSM health to provide instruction on how social determinants of health impact the care of GSM patients.	Statistically significant knowledge gains were noted in students in regard to achieving lecture objectives, for example, how sexual/gender minority status affects health care outcomes for patients, and how other social determinants of health such as race, socioeconomic status, and gender intersect with this issue.	Didactic lectures like this could be incorporated into a longitudinal curriculum on GSM health. Additional efforts are needed on faculty development and training on GSM health, and increasing comfort in faculty teaching this topic.
2	Eriksson and Safer^39^	43 of the 121 first year MS exposed to the curricular content responded to the pre-/post-audience survey, whereas 56 responded to the pre-test examination and 121 completed the post-test survey.	2 studies were conducted with the same sample. Study 1 consisted of a one-question anonymous survey regarding assessing student knowledge of the etiology of gender identity before and after content exposure. Study 2 consisted of a pre- and post-examination style study with two questions assessing student knowledge of gender identity, transgender medicine, and whether cross-sex hormone therapy is a valid treatment.	A single lecture on the biologic evidence for the durability of gender identity was added to a mandatory first year curriculum.	Regarding etiology of gender identity, there was a significant increase of correct responses. Regarding cross-sex hormone therapy, there was also a significant increase in correct responses	Brief didactic exposure to evidence-based gender identity training is associated with increases in knowledge. The small number of participants in the samples for both studies limit the generalizability of the findings
2	Gavzy et al.^40^	178 first year MS received the training, and 93% (166) completed both the pre- and post-surveys.	The Kern model was applied to design, implement, and evaluate the curriculum. This approach includes conducting a needs assessment to inform goals and objectives for the training, applying multimodal teaching methods to reach the goals, and finally collecting evaluation data and feedback from participants.	A 2.5-h workshop was delivered addressing the dimensions of human sexuality; personal experiences regarding sexuality and homophobia/transphobia; health issues faced by GSM-identified individuals; and provider-patient interactions. Graphics, didactic lectures, sexuality surveys, videos of patient interactions, as well as small group discussions were used to address the topics.	Data from 2017 to 2018 sexuality surveys indicated most students identified as straight and were open and comfortable with discussing sexuality. Students indicated a high degree of satisfaction with the sexuality survey, small group video discussions, didactic training, and the graphics used to teach the dimensions of sexuality. Student self-reported confidence in understanding GSM-related terms; identifying unique health care needs of GSM patients; and developing better practices working with GSM patients were all significantly higher at post-test than pre-test.	The multi-modal training experience was viewed favorably. The involvement of GSM students in the development of training was particularly helpful. This involvement had an ancillary gain of increasing a diverse leadership group within the school. Ensuring confidentiality on the sexuality surveys was key to the high response rates.
2	Greene et al.^41^	23 primary care residents	OSCE of a trained transgender standardized patient interested in having an orchiectomy and also presented with hypertension and hyperkalemia issues	Case-specific guidelines for transgender health were employed. Content includes discussing sexual activity and associated risks, making a plan for treatment considering the patient's health issues, and her values regarding hormones.	The communication and patient satisfaction scores exceeded 85%. Less than 61% of the residents made the patient feel comfortable and asked about her identity. 25–39% of the residents discussed treatment for high potassium and hypertension, respectively.	Good communication skills helped residents to improve their transgender-specific knowledge. Feedback from faculty and standardized patient helped residents how to ask about transition, and use of appropriate terminology. The need for including a transgender case in OSCE is recognized as a critical need to fill the curriculum gap in this area.
2	Grosz et al.^42^	167 first year MS were exposed to the content, and 73 matching pre- and post-test assessments were collected (43.7%).	Anonymous pre- and post-session assessments were administered, which assessed student GSM health knowledge and confidence in providing care.	2-h session consisting of a student-delivered presentation, patient-panel, and small group discussion. Content included GSM demographics, relevant terminology, legal issues, disparities, history taking, and gender transition.	Students' preparedness and comfort in providing care to the GSM group showed significant improvement.	The intervention session improved students' knowledge and confidence to provide care; however, the response rate to the survey was low.
3	Jin and Dasgupta^43^	All of the 180 year-1 MS exposed to the training content participated in in-session survey, while only 35% and 17% completed the pre- and post-test, respectively.	Assessment of students' ability to apply population genetics concepts was conducted during the session using mandatory audience response questions. Voluntary pre- and post-tests were collected to analyze changes in student attitudes and knowledge of assisted reproductive technologies for providing health care to same-sex couples.	A 1.5-h flipped classroom session that covered basic science population genetics, cisgender LGB cultural issues, and reproductive endocrinology followed by facilitated discussion. Students also read a book and watched a video in preparation for the course.	Students showed a high level of knowledge of population genetics concepts. After the session, student perceptions of same-sex couples compared to heterosexual couples changed and both groups were treated the same afterward.	Lack of incorporation of materials for transgender patients and their assisted reproduction needs is one of the recognized shortcomings. Live clinical encounters with LGB patients to facilitate consideration of the barriers faced by these patients were recommended.
3	Kelley et al.^44^	75 of 143 second year MS completed pre- and post-test surveys.	Pre- and post-test questionnaire to assess student knowledge, attitudes, and experience regarding a range of GSM health issues.	Training consisted of a 1-h patient panel, and a 1-h small group session focusing on 3 different case studies. The goal of the training was to increase awareness of student assumptions regarding GSM patients, highlight LGBT health disparities, and illustrate the physician role in improving LGBT health care.	While improvement was seen on most items from pre- to post-test, only 4 of the 16 items improved significantly, two regarding GSM knowledge and two related to attitudes about working with GSM patients.	This short, focused intervention resulted in moderate positive change in knowledge and attitudes regarding work with GSM patients. The low response rate, and lack of a longer term follow-up limit generalizability and do not indicate whether knowledge changes are durable.
2	Kidd et al.^45^	22 residents in psychiatry	A professionalism workshop on transgender health	A 90-min workshop was conducted to assess the resident's ability to empathize with and professionally treat transgender patients. Pre-test, post-test, and 90-day follow-up surveys were conducted to assess empathy, knowledge, comfort, skill, and motivation for learning.	65% of the residents completed pre- and post-surveys. 91% of these completed the 90-day follow-up survey. Post-workshop surveys revealed statistically significant increases in all the tested domains. No significant changes were found on a 90-day follow-up in any of the tested domains.	Significant short-term increases were found in professionalism toward transgender patients. Extended follow-up results reveal the limitations of one-time intervention. The authors' findings call the need for recurrent workshop programs to yield long-lasting improvements in knowledge and care.
2	Leslie et al.^46^	A total of 653 students participated in the study, of which 158 were first year MS.	Pre- and post-test design were employed measuring GSM self-reported knowledge and readiness for interprofessional learning.	A 75-min interdisciplinary training session, including personal stories from the GSM population; didactic lecture; an interprofessional case study; and debrief.	Significant improvement in knowledge and interdisciplinary learning readiness was reported. Analysis case study action plans indicated that half of the teams listed referral to a GSM specialist was the top solution for treating the patient. Participants also had more difficulty developing an action plan for a case study describing a questioning teen, than with a transgender woman.	While knowledge and self-rated skill were significantly higher from pre- to post-test, the action plans associated with the case-studies indicated a disconnect between knowledge and actual skill. This could be due to the students being in pre-clinical years and not having experience working directly with patients.
2	Marshall et al.^47^	85 of 163 first and second year MS filled out the overall course evaluation (52% response rate).	Post-test only cross-sectional survey consisting of one question: are you the same, less, or more prepared to work with GSM patients after attending the course.	A three and a half hour training consisting of didactics, small group exercises, and patient panels was provided. Content covered included common terms; etiology and complications of medical and surgical interventions; and addressing health care needs and disparities faced by transgender patients.	Single question survey indicated more preparedness of students to care for transgender patients. Positive qualitative responses regarding the course were also reported.	Responses to the question regarding preparedness were positive, as were the qualitative responses that were reported. The response rate was low, and the survey used to evaluate the course was not robust.
2	Mayfield et al.^48^	84 year-2 and year-3 MS participated in the study.	Pre- and post-tests were conducted to assess the preparedness of MS to take inclusive, comprehensive sexual histories from patients of all sexual orientations and gender identities	Training consisted of a 30-min large-group lecture and three 40-min small-group standardized patient encounters with debriefs. Pre-session tasks included watching a short video on sexual history taking, assigned readings, and an implicit bias activity.	Students showed a statistically significant improvement in comfort with their ability to take a sexual history in general, and also from patients with a differing sexual orientation.	This intervention resulted in improved sexual history taking skill among pre-clinical MS. Relatively brief interventions that include actual skills practice with standardized patients can be effective in skills acquisition. Longer term follow-up is needed to determine the durability of these changes.
3	McCave et al.^49^	494 students participated in the IPEC, of which 83 were MS. Additional student disciplines included nursing, occupational, and physical therapy, physician assistants, social work, and health care administration.	A post-test only design was employed assessing student's perceived value of the training to work with transgender patients and preparedness for practice.	Training consisted of a speaker panel of transgender community members; a simulation exercise of an interdisciplinary huddle to establish a care plan for a transgender patient; and a large group debrief.	Participants found the speaker panel most valuable, followed by the discharge planning meeting, the team huddle, the team debrief on the video simulation, the simulation itself, and finally the large group debrief. All activities were viewed as valuable by at least 60% of the participants. Participants reported high levels of preparedness regarding values and ethics; roles and responsibilities; interprofessional communication; and team work. A range of 84–93% reported being prepared in these areas.	Activities that included direct interaction with transgender people were ranked the most valuable, indicating interaction as a key to successful training. Interaction between the student disciplines was also noted as a valuable learning experience. Students reported learning from each other. Authors note that finding simulated patients who actually are transgender is an important factor in the success of the simulation.
2	McGarry et al.^50^	37 internal medicine residents	A seminar on self-reported level of preparedness and comfort in dealing with lesbian and gay patients.	A 3-h seminar, which included a video, didactic lecture, and case discussion, was conducted. Pre- and post-seminar surveys were conducted to measure preparedness and comfort in dealing with health care, psychosocial, and sexual issues of lesbians and gay men.	100% of the residents recognized the need for lesbian and gay care issues. 95% of the residents felt more prepared to care for these patients after the seminar. 66% of the residents reported an increase in comfort level after the seminar. 68% reported receiving education about lesbian and gay health issues in medical school. 89% of the residents reported treating lesbian and gay patients or having individuals from these groups as family members or friends.	Overall, the findings were promising. Educational interventions like this seminar are necessary to have lasting changes in preparation and comfort of residents dealing with patients to improve patient-physician interactions.
2	Neff and Kingery^51^	144 of 155 first year MS completed both the pre- and post-test assessments.	Pre- and post-test of knowledge regarding androgen insensitivity syndrome.	Participants engaged in two, 2-h training sessions using a PBL approach. The case used for the PBL exercise describes an adolescent female experiencing androgen insensitivity syndrome.	Answers on the post-test knowledge survey were both higher, and the distribution of answers was more consistent from pre- to post-test. Open-ended question responses indicated students had increased awareness of and sensitivity to DSD. The majority of respondents indicated they did not have any barrier to implementing their knowledge regarding DSDs.	The PBL approach lead to participants meeting their established learning objectives, resulting in better knowledge scores at post-test. The pre- and post-knowledge surveys did not ask the same questions, which precluded statistical analysis.
3	Park and Safer^52^	20 fourth year Boston University Medical School students taking a clinical elective course in transgender medicine participated in the study.	Pre- and post-elective surveys assessed student self-perceived level of comfort and readiness to care for transgender patients.	The intervention was a transgender medicine clinical elective where students rotated on services that provide clinical care for transgender individuals. The fourth year course built on education content delivered in years 1 and 2 on the biological basis of gender identity.	Students who reported “high” comfort with treating transgender patients increased from 45% to 80%. Students reporting “high” knowledge regarding management of transgender patients increased from 0% to 85% at post-exposure.	While integrating transgender-specific content into pre-clinical medical curricula improves student knowledge and comfort, gaps still remain. Clinical year exposure to transgender medicine can help close the gap and improve access to care for transgender individuals.
1	Potter et al.^53^	175 year-1 medical and dental students participated in the course from 2015 to 2016. Only 5 of 10 student representatives were included in the qualitative data collection.	Post-test only qualitative assessment of the intervention was conducted by surveying student participants and faculty facilitators.	The intervention session (2-h long) included pre-work, a didactic presentation, role-play scenarios, and a small-group session on talking to SGM patients about identity, intersectionality, and resilience. Faculty facilitators were provided training on how to run small-group and role-play exercises.	This training provided a unique opportunity to students to explore their unconscious biases. In addition, they were able to practice novel interviewing techniques in a clinical academic setting.	The students surveyed unanimously agreed that the topic of identity and intersectionality was an important addition to the curriculum. Some of the recommendations were to have a more structured small-group session and conduct role-plays about specific topics such as gender identity, sexuality, and race. The faculty surveys also revealed that the intervention session was successful in raising students' awareness about the health impacts of identity and intersectionality on SGM patients.
4	Safer and Pearce^54^	Boston University Medical School students in all 4 years participated. Year-2 student responses (74 pre-test and 66 post-test) were contrasted with the other years of medical school (183 pre-test and 168 post-test).	A prospective internet-based pre- and post-exposure survey was conducted assessing student comfort working with patients requesting natal female and male patients to become male and female, respectively, and female and male patients seeking care for hypogonadism.	Curriculum content was added to the endocrinology unit of a mandatory second year pathophysiology course. The content consisted of a single dedicated lecture that taught rigidity of gender identity, classic treatment regimens, and monitoring treatment regimens.	Post-training, there was a significant increase in the number of year-2 students reporting comfort treating transgender patients. None of the year-2 students agreed with the statement that hormonal treatment for transgender patients was not a part of standard care after exposure to the intervention.	Modest curriculum change can significantly increase students' self-reported willingness to care for transgender patients. However, comfort in treating transgender individuals was not significantly improved, indicating more training may be needed to improve this.
2	Salkind et al.^55^	433 MS pre-intervention and 541 MS in all years in a London-based medical school.	Pre- and post-exposure to a modified curriculum using a five-point Likert scale questionnaires were administered to assess course satisfaction, confidence in using appropriate terminology, and working with GSM+ patients.	The curriculum included three components: (1) A didactic session on background knowledge, terminology, GSM inequality, legal protection for GSM- people, and professional guidance; (2) a small group discussion with a transgender patient (3) a PBL exercise. The intervention was facilitated by self-identifying GSM+ junior doctors.	A majority of the students surveyed found the teaching (95%) and the self-identified GSM visitor's input (97%) helpful. Students' confidence using appropriate terminology was increased from 62% to 93% (*p*<0.001) and gender identity from 41% to 91% (*p*<0.001). Similarly, students' confidence in clinical assessment of a GSM patient increased from 75% to 93% (*p*<0.001), and of a transgender patient from 35% to 84% (*p*<0.001).	This teaching program developed and delivered in collaboration with the GSM community received a positive evaluation from students and was associated with increased student confidence using appropriate language related to sexual orientation and gender identity, and performing clinical assessments on GSM patients. Further studies also call the need for additional research to find out whether the improved student confidence translates into improved patient care for the GSM individuals.
3	Sawning et al.^56^	39 of 52 MS who attended at least one session of the certificate program participated in the study. Students from any year of medical school were eligible to participate.	Pre- and post-test assessing knowledge and attitudes regarding practice with GSM patients.	An 11-session certificate program covering the topics of leadership in GSM health, skills for medical practice with GSM patients, affirming care, health disparities, ethics and law, and mental health. Social cognitive learning theory informed the pedagogy.	Knowledge, attitudes, and skills regarding work with GSM patients increased significantly after attending the certificate program training. Increases in agreement that same-sex sexual behavior and transgender identities were natural expressions of sexuality and gender increased similarly from pre- to post-test.	The social cognitive theory model of training used in this study exposed students to role models of affirming care and provided opportunities to practice. Exposure to this model of training was associated with improved outcomes even among those who did not complete all modules. Furthermore, the course was developed using local experts with existing expertise, and as such was a low-cost intervention.
2	Streed Jr. et al.^57^	833 internal medicine residents in 120 internal medicine programs in the United states	A 1-h online LGBT health module addressing SGM health	A pre-test was conducted to assess residents' knowledge on SGM. A didactic module reviewing diagnosis and management of diseases was completed followed by a post-test. The tests evaluated residents on 4 aspects, which include (1) terminology relevant to SGM patients; (2) health disparities and preventive care issues affecting SGM patients; (3) substance use and mental health issues unique to SGM patients; and (4) common sexually transmitted illnesses affecting SGM patients.	There was no difference between level of training and overall pre-test- (ranged from 50% to 52%) or post-test (ranged from 80% to 82%) in performance of participants. The participants demonstrated an improvement between pre- and post-test knowledge.	Knowledge on health issues of SGM did not change during residency training. Improved physician training on clinical care of these vulnerable populations may improve care and reduce disparities in receiving care.
3	Thomas and Safer^58^	144 internal medicine residents and 29 family medicine residents were invited, 38 completed the needs assessment and 20 residents completed the pre-test and 21 completed the post-test.	Pre- and post-lecture surveys that assessed the residents' knowledge and willingness to assist transgender patients with hormonal therapy.	A 60-min lecture on transgender medicine covering the durability of gender identity and hormonal treatment regimens was given separately to the internal medicine and family medicine residents.	Significant increases in the percent of residents who felt sufficiently knowledgeable to assist with hormonal therapy for a female-to-male as well as male-to-female patients were found from pre- to post-evaluation.	The intervention significantly increased residents' knowledge and willingness to assist with hormonal therapy for transgender patients. Continuing medical education programs and professional conferences may be potential settings for interventions to increase transgender knowledge in physicians.
3	Thompson et al.^59^	136 first year MS completed the pre-test, and all completed the post-test in year 2 of medical school.	Pre- and post-assessments of knowledge of transgender and LGB populations, health care needs, and comfort level with patients. A free response question was also posed to the students regarding the knowledge and skills they would like to acquire, and these responses were analyzed using natural language processing.	Students attended a panel discussion with LGBTQ employee panelists, took and reflected on the implicit associations test, and were given the National Transgender Discrimination Survey Report. Students also watched the film Transgender Tuesdays, completed the Fenway Institute course on LGBT health disparities, HIV prevention, and providing care the LGBT patients.	Statistically significant improvement in gender identity competency, skills, and self-reported knowledge was seen from pre- to post-tests.	The curriculum change improved students' gender-affirming medical competency, knowledge, and skills.
3	Ufomata et al.^60^	100 of 153 internal medicine residents and 29 of 35 preceptors completed the pre-survey, and 57 residents and 14 faculty completed the first post-survey.	Pre- and post-test assessing knowledge and confidence. A cross-sectional administration of IAT was employed as an initial needs assessment.	A four-session training addressing knowledge of GSM issues, cultural competence, health promotion, disease prevention, mental health, violence, and reproductive health was provided. Pedagogy included didactics, case vignettes coupled with discussion, and provision of a reference list.	Overall confidence in knowledge of primary care with GSM patients improved. Some subsections of both knowledge and confidence increased significantly.	IAT scores indicated residents and faculty slightly favored straight over LGB persons. While knowledge and confidence increased, the number of students and faculty completing the post-tests was low. Content was taught by faculty without specific training in GSM health, indicating low-cost implementation.
1	Underman et al.^61^	64 fourth year MS received the intervention and completed the post-exposure survey.	Post-test survey and observational qualitative data were collected.	A transgender standardized patient case and 1-h overall debriefing led by a clinical faculty member. After the debriefing, students completed an evaluation of the case they worked on.	About 80% of students agreed or strongly agreed that the transgender patient scenario increased their knowledge and skills. MS lacked basic communication skills for interacting with transgender patients.	The standardized patient case can be adaptable to other formats to allow use by other schools. Future patient cases will need to be revised so MS can focus on the patient's reluctance to visit the doctor and not their access to life insurance.
3	Vance et al.^62^	20 learners, including pediatric interns, psychiatry interns, and year-4 MS.	Pre- and post-tests were conducted to assess pediatric trainees' and students' knowledge of the psychosocial and medical issues facing transgender youth.	The intervention included six interactive, online modules and an observational experience in a multidisciplinary pediatric gender clinic.	Statistically significant improvement was noted in knowledge and awareness of transgender-related health issues. Furthermore, knowledge/awareness scores rose from “not knowledgeable/aware” to “knowledgeable and aware” among 13 of the 20 student respondents.	The interactive online modules and observational experience in a pediatric gender clinic were well received by learners. Furthermore, the online delivery method coupled with an observational experience resulted in improved knowledge, indicating a promising delivery method for this content.
3	Vance et al.^63^	31 students, including year-4 MS, psychiatry interns, and nurse practitioner students at the University of California San Francisco participated in the study.	Pre- and post-exposure surveys were administered to assess the effect of an online educational intervention on transgender youth patient knowledge and perceived self-efficacy among participants.	The intervention consisted of only the six online modules described above in Vance et al.^62^ to parse the effect of the online training modules from the pediatric observational experience.	A statistically significant improvement was found in self-perceived knowledge of transgender patients, as well as self-efficacy regarding evaluation and counseling skills with transgender youth.	The assessment of the six online modules without the observational experience noted in Vance et al.^62^ resulted in significant knowledge and self-efficacy improvements, lending further promise to the utilization of online training.
3	Wahlen et al.^64^	107 of the 157 fourth year MS approached participated in the study. 86 (54.7%) completed both the pre- and post-test.	A pre- and post-exposure survey was used to evaluate the knowledge, attitudes, judgment, and experience in working with GSM.	The intervention included a single 1-h lecture on sexual orientation and gender identity development in adolescence, emphasizing health issues faced by GSM.	Overall, the intervention resulted in significant increases in knowledge, attitudes, judgment, and experience regarding GSM health issues.	While significant changes in all constructs from pre- to post-assessment are noted, the changes were modes in all categories, except knowledge. Furthermore, students who did not attend the lecture also reported similar increases in scores on all constructs. Scores on all four constructs were high at pre-test, and the proportion of GSM students in the study were high, either or both may account for the modest increases from pre- to post-test.

DSD, disorders of sexual development; GPs, general practitioners; HIV, human immunodeficiency virus; IAT, Implicit Association Test; IPEC, Interprofessional Education Collaborative; LGB, lesbian, gay, and bisexual; MS, medical students; OSCE, objective structured clinical examination; PBL, problem-based learning; SGM, sexual and gender minority.

### Quality assessment

The quality of each study was evaluated according to published recommendations^65^ using a rubric ranking article from 1 (low quality) to 5 (high quality). Each publication was assigned to two reviewers for independent ratings. When the ratings differed, the articles were assigned to an additional reviewer to provide a third perspective and help the original dyad reach consensus. Studies that used a nonexperimental or pre-experimental design were rated as 1 or “Weak Quality,” but this ranking also included studies that used only a qualitative or cross-sectional design. Studies that employed a pre-test/post-test design only without a control or comparison group were rated as a 2–3 or “Moderate Quality.”

Studies rated as 4 or 5 or “Strong quality” included a pre-/post-control design or a longitudinal design with a control group, coupled with assessments of changes in behavior/skills, a large sample size, or a high participation rate of students/residents. Studies were not excluded from the review based on research design; instead, their limitations (including lack of research rigor) were identified and rated.

### Data analysis and synthesis

Meta-analysis of the studies reviewed was precluded by the lack of heterogeneity of research designs, assessment measures, and samples. Instead, a qualitative analysis of the training approaches and related findings was conducted.

## Results

A total of 27,090 articles were initially identified with 36 remaining after 3 stages of review. Articles at this stage assessed the impact of educational evaluations in terms of course satisfaction, knowledge increases, changes in attitudes beliefs, trainee comfort when treating SGM patients, and finally skill acquisition.

### Study characteristics

Table 2 provides a summary of the studies included in this review. Studies reported sample sizes ranging from 5 to 833 participants. A total of 5840 combined observations were included across all studies. Of the 36 studies reviewed, 27 included a sample of medical students and interprofessional cohort of students (i.e., nursing, social work, physical therapists, and health care administration students) only, 6 articles sampled residents only, and 3 assessed a combination of medical students and residents. The educational/training methods used a variety of methodologies, including didactic sessions, patient panels, standardized patients, small group discussions, and student-delivered presentations.

The duration of trainings described in the studies ranged from 1 h to 10 weeks. Pre- and post-test designs were by far the most common research strategies used in the studies (*n*=31), followed by post-test only evaluation (*n*=4). One study using the Kern model, a six-step curriculum development approach, which ends with an evaluation of the developed curriculum. The quality ratings of the studies are summarized in Table 2.

### Quality ratings of studies

Studies included in the review had an average research quality rating of 2.4 on a five-point scale. Although the majority of studies in the review included both pre- and post-exposure evaluations, some studies contained no control or comparison group or only post-exposure evaluations. These were generally rated at a “2” due to threats to validity. One study was rated at a “4” owing largely to the use of a natural comparison group.^54^ Three studies received a “1” rating due to the use of a post-test-only design (no control group) or a poor description of the research methodology.^53,59,61^

No study received a rating of 5 as none utilized rigorous study designs. While the study ratings were moderate to low regarding study design quality, we included all articles as these types of designs are not uncommon in educational settings and capturing all the approaches to affirming care training was an important part of this project.

### Pedagogical methods employed in the study

Didactic training was the most frequently used pedagogical method, either as a stand-alone strategy (*n*=6) or in combination with other methods (*n*=19). The next most commonly used approach was group discussion, both in small and large groups (*n*=17); in all cases, these interventions were combined with one or more other methods. Patient panels (*n*=12) and case study reviews (*n*=9) were commonly used as well, but always in combination with other approaches. Mock interviews with standardized patients or with fellow students/residents were employed in eight of the studies.

Four studies reported training outcomes of clerkship rotations as well as online modules. A problem-based learning activity was used in two studies and the remaining study included a student-led presentation. The phrase “Sexual and Gender Minority (SGM)” was used to describe patients in two of the studies,^31,38^ in contrast to different versions of “LGBTQ+” in the remainder of the articles.

Seven assessment domains and 9 pedagogical approaches were identified across the 32 studies, but a study comparison (or meta-analysis) was not feasible because each study employed varying combinations of pedagogy and evaluation. However, multiple pedagogical approaches were found to be more effective among the studies that reported significant positive changes on multiple measurements combined. For instance, the Berenson et al.'s^33^ study assessed knowledge regarding transgender health disparities and increasing confidence in providing hormone therapy to transitioning patients in second year medical students after providing content through a combination of didactic lectures, patient panels, and small group discussions. The authors found significant gains in both domains with similar results matching multimodal approaches to multiple assessment domains had similar positive outcomes.^33,40,56,60^

Of the four interventions that included a clerkship rotation or resident rotation, three reported significant positive changes on a multiple assessment axis. Bakhai et al.^32^ reported significant increases in knowledge, comfort, and skills in communicating and conducting physical assessments with SGM. Park and Safer^52^ reported similar findings, significantly increasing knowledge confidence, and attitudes in working with transgender patients. The two remaining studies of rotation evaluation reported positive increases in two domains relative to SGM patients (knowledge and confidence).^63,66^

Time allotted for training ranged from 1 to 11 h (excluding rotations that lasted for 4 or 8 weeks). While some curricula with longer durations (>9 h) had positive outcomes in more than one measurement domain,^34,56^ others did not.^29,67^ Several of the shorter trainings (<10 h) resulted in improvement on multiple evaluation axes.^30,33,36,37,39,42,44,45^

## Discussion

Our systematic review summarized the findings of 36 training interventions (UME and GME) and identified training approaches with positive outcomes in regard to increased knowledge, comfort, and skill, as well as improved attitudes of students in working with SGM patients. Satisfaction with SGM training materials was also reported in our review. In general, students reported high levels of satisfaction with SGM training material. Limited time allotted to gender-affirming care module in the curriculum is one of the constraints as learners will have less protected time to complete the module.^38,63,66^

Therefore, several studies recommended an increase in the amount of time that is dedicated to the delivery of affirming/inclusive care to SGM patients in both the UME and GME curricula.^29,32,34,35,42,44,45,57^ Furthermore, long-term follow-up to measure the efficacy of curricular interventions (retention of knowledge, skills, comfort, confidence, attitudes, etc.) and make the interventions generalizable to other health care professions is another barrier for inclusive and affirming care training during UME.^38,60–63,66^ The impact of curricular interventions on various domains is given below.

### Impact of interventions on satisfaction

We also assessed participant satisfaction with the training interventions for our systematic review. Five studies that assessed satisfaction utilized 5-point Likert scale surveys as is typical of course evaluations,^31,33,40,49,55^ while one used a 10-point scale.^67^ All the interventions received generally high or positive ratings by participants in terms of satisfaction, with two studies reporting 90% or higher satisfaction rates.^49,55^ These evaluation scores reflect high student satisfaction with affirming care training. High reported satisfaction (seeing the value in knowledge) is an important prerequisite to knowledge construction in that, it is an indication of student engagement (response rate).^32,42,59^

### Impact of interventions on knowledge

Knowledge was the most frequently assessed outcome of affirming and inclusive care training. Short duration, didactic training in our review was shown to be effective, as well as multimodal approaches delivered over longer periods. Twenty-six of the articles reviewed included an assessment of student/resident knowledge acquisition. The measurement of knowledge covered several domains, including clinical knowledge, cultural competency regarding SGM patients, health disparities that SGM patients face, and health policies affecting SGM patients. Nine of the articles focused on knowledge acquisition about SGM. Twelve of the articles measuring knowledge focused on transgender patients.^30–33,38,43,61,63,64,66–68^ Two articles examined knowledge acquisition regarding hormone therapy for transgender patients.^39,56^ Both reported significant knowledge gains. With one exception, the studies evaluating knowledge increases indicated significant positive improvement from pre- to post-test, save 1, in which only 2 of 16 knowledge measures improved from pre- to post-test.^44^

### Intervention impact on attitudes and beliefs

Of the articles reviewed, eight included assessments of changes of attitudes and beliefs relative to care of SGM patients.^29,34,36,43,44,53,56,64^ Six of the interventions targeted medical students only, primarily those in the pre-clinical years (years 1 and 2), while one intervention targeted resident physicians only^58^ and one a combination of residents and students.^36^ Delivery modalities varied across each intervention; however, most of the interventions were incorporated into existing curricula within the learning program.

Six of the studies highlighted interventions that focused on student/resident attitudes toward SGM health issues. Although knowledge about gender-affirming care increased, improving student attitudes regarding working with SGM patients proved to be a more difficult task in the studies we reviewed. One of the articles reported no significant change in attitudes regarding SGM health after the intervention.^29^ Another demonstrated positive attitude changes regarding work with same-sex couples,^43^ and two articles reported significant increases in general attitudes toward working with SGM patients.^56,64^

Kelley et al. found only a slight improvement in student attitudes regarding work with SGM patients.^44^ Finally, one article that used a qualitative assessment of student attitudes found students appreciated the opportunity to discuss unconscious bias regarding working with SGM patients.^53^ A final article not only reported improvement in attitudes toward sexual behavior of SGM patients but also reported decreases in student attitudes toward conducting sexual histories with SGM patients.^34^

The two articles addressed attitudes in working with transgender patients. The first found significant decreases in transphobic attitudes post-intervention.^34^ Cherabie et al. conducted the only intervention that extended beyond student learners and included resident faculty.^36^ Overall, there was a significant improvement in attitudes toward transgender health issues when comparing the pre- and immediate post-intervention surveys. Of the three studies that did report significant changes in both attitudes and beliefs, two were longer than 10 h in duration,^34,56^ while one was 2 h in duration and included multiple pedagogical approaches (patient panels, case studies, and small group discussions).^44^

However, these significant gains were not maintained at a survey conducted 90 days post-intervention. The one study that examined longer term outcomes (90 days) indicated that attitude improvements may not be maintained across time.^36^ These findings suggest that training intended to improve attitudes or challenge problematic beliefs among trainees may need to be longer and utilize more interactive approaches and direct exposure to SGM. More studies are needed, however, to clarify the critical elements of medical training that lead to improved attitudes and to examine reasons for the attrition over time, as in the one study that reported an extended assessment, most of the attitude change noted at immediate post-training assessment was lost at the 30-day follow-up assessment.

### Impact of interventions on physician comfort

Few studies have evaluated the impact of training interventions on improving the comfort level of medical students working with transgender clients.^36,44,52,54^ All the interventions that measured comfort resulted in significant improvement post-training.^32,36,37,48,52,54,58^ Interventions have included delivery of didactic content, usually providing information about transgender experiences, delivery of care and hormone regimens,^36,52,54^ and clinical experience. Only two^36,37^ of these seven studies reported using a didactic approach as the sole pedagogical method employed, while the others reported patient panels, rotations, small groups, and standardized patients. For example, Sawning et al. examined the effect of added training in a fourth year Transgender Medicine elective, with students exposed to transgender patients seeking medical and surgical treatment.^56^

At the Boston University School of Medicine, a dedicated lecture focused on treatment regimens and monitoring therapy for transgender people was given to second year students during their endocrinology unit. Questionnaires were administered three times. The first survey was given 1 month before the lecture and two surveys were administered 1 month and 90 days after the lecture. Before the lecture, 38% of second year students expressed discomfort at the idea of providing transgender care for a patient. Immediately after the training, this decreased to 12%.^54^

At East Tennessee State University, participation in a half-day integrated grand rounds, including presentations by basic science and clinical faculty, patient presentations, and small group discussions, resulted in improved comfort interacting with transgender patients, increased knowledge base for providing care to the transgender population, and reduced the preference not to treat transgender people. Students also had improved scores on approval of the single statement “learning from transgender patients will help me be a culturally competent medical student and future physician.”^36^

A 1-h didactic lecture on transgender medicine at the University of Kansas School of medicine included presentations by transgender people to faculty, residents, and medical students. This was evaluated with pre- and post-surveys (one immediately following the lecture and another at 90 days). Comfort levels significantly increased from pre- to post-survey and remained high at 90 days. Questions included “I feel comfortable using language that respects gender identity” and “I feel comfortable discussing options for gender confirming hormone therapy.”^36^

Only one study evaluated change in comfort after a clinically based intervention. In the Boston University School of Medicine medical elective in transgender medicine for fourth year medical students, students were given pre- and post-elective surveys that included the question, “What is your level of comfort with providing care to a transgender patient?” Although this was a small sample size, the percentage of medical students who reported a high level of comfort providing care for transgender patients significantly increased from 45% (9/20) to 80% (16/20) post-intervention.^52^

While training strategies that included exposure to SGM persons or being exposed to the beliefs of others as happens in small group discussions might hold particular promise in improving comfort, our findings are not conclusive on this point, but do support further study of these interventions.

### Impact of interventions on confidence/readiness

All the studies that measured confidence post-training reported increases.^30,31,33,40,42,47,52,55,66^ The studies were delivered at different points in student training as well as in graduate medical training. Various modalities ranging from classroom presentations, workshops, patient panels, online training sessions, including videos, were used across the different studies and all resulted in self-rated improved confidence.

One study focused on providing transgender health care for adults, adolescents, and children.^30^ The intervention included a 1-h educational session conducted by a member of the transgender community targeting third year medical students, general practitioners, and internal medicine physicians. The content delivered in the interventions included transgender terminology, exploring the biological basis (genetic, genital, and neurological) of gender identity and diversity, the lived experience of a transgender individual, and their relationship with health care providers, supportive care for children, and other family members, adolescent puberty blockade, adult transition care, fertility options, and hormonal monitoring and surgery. The follow-up post-intervention survey revealed increasing confidence among participants in administering health care to transgender individuals.

In a similar manner, the Salkind et al.'s study incorporated a visit from a transgender patient in a mandatory teaching program on SGM health.^55^ This educational intervention developed and delivered in collaboration with a representative from the SGM community was found to enhance students' confidence in using appropriate terminology during SGM patient encounters and also for clinical assessment of patients from these vulnerable populations.

Aside from faculty, student-driven interventions also yielded fruitful results. One of the student-led intervention studies was aimed at enhancing the sexual history-taking skills of the first year medical students.^31^ The intervention was developed through collaboration between faculty and students. The intervention module that was integrated in the clinical skills course consisted of three modules: an e-lecture (14 min) on sexual history taking, a standardized patient interview (35 min), and a debrief (20 min) of standardized patient activity. The assessment appeared to improve confidence among students to tackle sexual orientation and gender identity after intervention.

Another student-led intervention (by second and fourth year medical students) was developed to enhance the understanding of first year medical students about SGM health issues.^42^ The intervention session included a presentation, patient panel, and small group discussions. The intervention resulted in significant gains both in student knowledge of SGM and in confidence in providing care to SGM patients. Of the three intervention modalities, the patient panel was greatly appreciated by the participants as it helped them understand the clinical perspective. A similar student-led intervention approach was evaluated that included a classroom presentation on transgender health disparities, a small-group session viewing a physician, transgender patient communication, and a large group transgender patient-panel.^33^ These student surveys also indicated greatly improved student confidence caring for transgender patients.

Although most of the studies involved more advanced students, an early introduction to SGM health in medical education also appears to benefit medical students. The impact of an interactive multimodal workshop primer on SGM health comprising a PowerPoint presentation, sexuality survey, videos of provider-patient encounters, and community-based resources given to first year medical students was assessed in one study.^40^ Student evaluations showed an increase in confidence comprehending multiple aspects of human sexuality, health issues for SGM patients, and promoting affirming care.

Another study sought to improve the clinical care of transgender patients through longitudinal curricular changes by adding a section on gender identity in an M1 Physiology course coupled with sessions on transgender hormone management in the M2 Endocrinology course and M4 clinical elective.^52^ Post-intervention surveys revealed a significant improvement in students' knowledge and confidence regarding management of transgender patients. An additional study demonstrated an increase in confidence and readiness of students to care for transgender patients after introducing a didactic component on basic science principles applied to transgender patients and then staging a mock encounter between a health care provider and a patient with gender dysphoria.^47^

Very few studies have focused on training received on SGM health care in GME curricula. In one study, an intervention was evaluated for fellows and learners in post-graduate medical education.^67^ The intervention included the following four curriculum modules: (a) cultural humility introduction and patient introduction; (b) gender and sexual orientation identity development; (c) discussion of gender dysphoria and transgender youth health using a new patient case study; as well as a debate to explore SGM-related health and social policy issues. The learners expressed increased confidence in identification of community resources, understanding of cultural humility, adolescent health, and interprofessional training and collaboration.

In a novel application of the Implicit Association Test, assessing one's personal implicit bias toward SGM patients was employed as part of a curriculum with study objectives taken from the AAMC and the Fenway guide to SGM health.^60^ The internal medicine residents who took this course demonstrated increased knowledge and confidence in providing care to SGM patients.

Pediatric and psychiatric transgender care also require an additional array of expertise and skills. Vance et al. focused on enhancing the effect of training modules on self-perceived, objective knowledge, and clinical self-efficacy of pediatric interns, psychiatry interns, M4 medical students, and nurse practitioner students.^63^ The intervention included transgender curriculum training at different intervals for a month. Post-tests showed that online learning was an effective intervention tool to enhance transgender-related knowledge and confidence in providing care to SGM patients. All the aforementioned studies yield promising findings, but as confidence is often used as a proxy measure for actual skill acquisition, further studies are needed to determine the correlation of self-reported confidence and actual skill.

### Impact of intervention on skills/competency

Overall, six studies measured the impact of the intervention on student skill level treating SGM patients.^32,44,46,59,61,62^ One study used a standardized patient to measure student's skill acquisition treating SGM patients.^61^ In this study, 80% of students agreed that the curriculum increased their skills for working with transgender patients. All six of the studies showed significant increases in participant skills; however, five^32,44,46,59,62^ of the studies' main measure of skill was participant knowledge and awareness of the skills necessary to treat SGM patients, not direct skills measurements. Standardized patients' interviews were used as part of the training in the Bakhai et al.'s 8-week pediatric clerkship study, but again no objective measure of how those skills improved was assessed, only self-reported preparedness on the skills necessary.^32^

An appraisal of various training domains revealed that for the conservation of valuable curriculum time, identifying training approaches that achieve desired goals is critical and early foundational exposures to affirming and inclusive care might well be accomplished through short didactic training. This is an area for further study, as an actual improvement in physician skills is the prerequisite to improved care for SGM patients.^68–70^ Studies that examine student and resident skill objectively are sorely needed in the affirming and inclusive care training literature.

### Limitations

This review provides a menu of potential measurement approaches for student knowledge, attitudes, comfort, confidence, and most importantly skills that can be used in further studies having a rigorous (with enough statistical power) experimental design. We did not identify any study that used either cross-sectional or longitudinal or case–control or randomized or retrospective experimental designs to evaluate the impact of affirming and inclusive care training modalities on knowledge, comfort, and skills of trainees in UME or GME.

## Conclusion

Several effective training approaches were identified, which improved student knowledge and attitudes toward providing affirming and inclusive care for SGM persons. Attitude changes, however, were found to require more interactive exposure to the SGM population. This review did not identify any strategy for improving attitudes and change beliefs toward SGM persons over time. Results suggest that comfort, confidence, and skills in providing inclusive and affirming care are better achieved through practice (clerkships and rotations) or mock practice (role play, case studies, and standardized patients) than didactic approaches only.

There is a clear need for more rigorous study of affirming and inclusive care training that assesses changes in knowledge, attitudes, and skills over extended periods beyond post-training. In addition, there is a clear need to include affirming and inclusive care training in UME, GME, and allied health professions. In addition, there is a need to assess training effectiveness of teaching students to provide affirming care to SGM persons on patient outcomes. There currently is a dearth of such studies in the published literature.

## Abbreviations Used

AAMC: Association of American Medical Colleges
DSD: disorders of sexual development
GME: graduate medical education
GPs: general practitioners
HIV: human immunodeficiency virus
IAT: Implicit Association Test
IPEC: Interprofessional Education Collaborative
LGB: lesbian, gay, and bisexual
MS: medical students
OSCE: objective structured clinical examination
PBL: problem-based learning
SGM: sexual and gender minorities
UME: undergraduate medical education
